# Large-scale mitochondrial DNA analysis reveals new light on the phylogeography of Central and Eastern-European Brown hare (*Lepus europaeus* Pallas, 1778)

**DOI:** 10.1371/journal.pone.0204653

**Published:** 2018-10-04

**Authors:** Mohammad Reza Ashrafzadeh, Mihajla Djan, László Szendrei, Algimantas Paulauskas, Massimo Scandura, Zoltán Bagi, Daniela Elena Ilie, Nikoloz Kerdikoshvili, Panek Marek, Noémi Soós, Szilvia Kusza

**Affiliations:** 1 Department of Fisheries and Environmental Sciences, Faculty of Natural Resources and Earth Sciences, Shahrekord University, Shahrekord, Iran; 2 Department of Biology and Ecology, Faculty of Sciences, University of Novi Sad, Novi Sad, Serbia; 3 Institute of Animal Husbandry, Biotechnology and Nature Conservation, University of Debrecen, Debrecen, Hungary; 4 Department of Biology, Faculty of Natural Sciences, Vytautas Magnus University, Kaunas, Lithuania; 5 Department of Veterinary Medicine, University of Sassari, Sassari, Italy; 6 Institutes for Agricultural Research and Educational Farm, University of Debrecen, Debrecen, Hungary; 7 Research and Development Station for Bovine Arad, Academy for Agricultural and Forestry Sciences, Arad, Romania; 8 Tbilisi Zoo, Tbilisi, Georgia; 9 Polish Hunting Association, Research Station, Czempiń, Poland; National Cheng Kung University, TAIWAN

## Abstract

European brown hare, *Lepus europaeus*, from Central and Eastern European countries (Hungary, Poland, Serbia, Lithuania, Romania, Georgia and Italy) were sampled, and phylogenetic analyses were carried out on two datasets: 1.) 137 sequences (358 bp) of control region mtDNA; and 2.) 105 sequences of a concatenated fragment (916 bp), including the cytochrome *b*, tRNA-Thr, tRNA-Pro and control region mitochondrial DNA. Our sequences were aligned with additional brown hare sequences from GenBank. A total of 52 and 51 haplotypes were detected within the two datasets, respectively, and assigned to two previously described major lineages: Anatolian/Middle Eastern (AME) and European (EUR). Furthermore, the European lineage was divided into two subclades including South Eastern European (SEE) and Central European (CE). Sympatric distribution of the lineages of the brown hare in South-Eastern and Eastern Europe revealed contact zones there. BAPS analysis assigned sequences from *L*. *europaeus* to five genetic clusters, whereas CE individuals were assigned to only one cluster, and AME and SEE sequences were each assigned to two clusters. Our findings uncover numerous novel haplotypes of Anatolian/Middle Eastern brown hare outside their main range, as evidence for the combined influence of Late Pleistocene climatic fluctuations and anthropogenic activities in shaping the phylogeographic structure of the species. Our results support the hypothesis of a postglacial brown hare expansion from Anatolia and the Balkan Peninsula to Central and Eastern Europe, and suggest some slight introgression of individual haplotypes from *L*. *timidus* to *L*. *europaeus*.

## Introduction

The brown hare (*Lepus europaeus* Pallas, 1778) is a native species to Northern, Central, Western Europe and the Western part of Asia, and it was introduced as a game into several countries (Argentina, Australia, Barbados, Brazil, Canada, Chile, Falkland Islands, New Zealand, Rèunion and the United States; [1]).

The effect of translocation on hare genome was proved by previous genetic studies and they suggested that the brown hare and the Cape hare (*Lepus capensis*) are the same species [2]. However, later the same authors performed mitochondrial DNA (mtDNA) analysis and found a significant divergence between them, and therefore they are currently considered to be two different species [3]. Pierpaoli et al. [4] showed that Italian and European hares did not share any mitochondrial haplotypes, indicating the lack of interspecific gene flow between the two species due to reproductive isolation in the course of their long separate evolutionary history. They identified two main groups of Eurasian and African hare haplotypes: Clade A (*L*. *granatensis*, *L*. *corsicanus*, *L*. *timidus)* and Clade B (*L*. *c*. *mediterraneus*, *L*. *habessinicus*, *L*. *starcki*, *L*. *europaeus*). These results suggest that the three species belonging to Clade A, with a common ancestor, would have colonized Europe independently of *L*. *europaeus* and would have originated by isolation during the Pleistocene glaciations in the southern or northern areas of refuge.

It is strongly argued that the current geographical distribution of temperate species and genetic relationships among their populations have been influenced by the climatic oscillations during the Late Quaternary [5, 6]. Specifically, different lineages represent populations repeatedly isolated into distinct glacial refugia such as the Iberian, the Apennine, the Balkan Peninsulas and Turkey [5, 7–10]. Furthermore, different human activities, competition for food or breeding and hybridization between species also led to a higher diversity in the southern refugial areas and the present genetic diversity of the hares [11–13]. There is evidence for human-mediated translocations that is well documented in the southern part of Europe [14].

Previous studies based on mitochondrial DNA (mtDNA) analysis on extant brown hare populations has revealed a relatively high degree of geographic partitioning [6, 15–18]. These studies distinguished two major geographically distinct lineages, the European (EUR) and the Anatolian/Middle Eastern (AME) clade. The EUR lineage is further subdivided into two subclades: the Central European (CE) and the South-Eastern European (SEE) [6]. The CE subclade includes individuals from across North-Central Europe, whereas the SEE comprises hares living in South-Eastern Europe. The second lineage, AME, includes individuals from Anatolia, South-Eastern Europe and the eastern Mediterranean Islands [17].

A recent study [18] found that there were three major haplogroups including Anatolia/Middle East (AMh), Balkans (BLh), and central Europe (cEUh) among brown hare populations worldwide. Additionally, three subgroups were revealed within the BLh haplogroup including South-Eastern Balkans (SEB), Southern Balkans (SB) and Greek islands excluding those harboring A-lineages (GI-B) off the Anatolian coast. Moreover, the South-Eastern and Central Balkans (SEB), comprising northeastern Greece, south and North-Western as well as South-Central Bulgaria, north-eastern part of Republic of Northern Macedonia, South-Eeastern and South-Western Serbia, was identified as the primary source region for most other Balkan brown hare populations [18].

On the other hand, Anatolian/Middle Eastern haplotypes have not been observed in South, Central and North-Western Greece and the rest of Europe, with the exception of one Serbian haplotype [18]. Also, European haplotypes have not been reported across the entire species range in the Middle East [6, 15, 19]. Further, the existence of a contact zone between the European and Anatolian/Middle Eastern lineages was detected in Bulgaria and North-Eastern Greece [6, 10, 15].

Detection of brown hare lineages is mostly based on the mtDNA control region (CR), and is usually well supported by cytochrome *b* (cyt *b*). It proves that mtDNA genomic data are useful in determining phylogenetic relationships between closely related species and within species [20–21] and for understanding the extent and nature of contact zones [10].

Overall, despite a relatively large number of genetic studies on brown hares, their phylogenetic relationships still remain challenging. Only several broad-range studies of phylogeography of brown hares have been done, relying on mtDNA control region sequences from Serbian, Greek and Bulgarian hares [6, 15, 18, 22–26]. Using wide-range geographic sampling over seven countries, we aimed to study (i) the extent of mitochondrial genetic variability and diversity of the brown hare in Central and Eastern Europe; (ii) the phylogeographic relationships of the studied populations, and furthermore (iii) to provide comprehensive information on the genetic characteristics of brown hares for conservation programs and management plans.

## Materials and methods

### Sample collection

**Fig 1:**
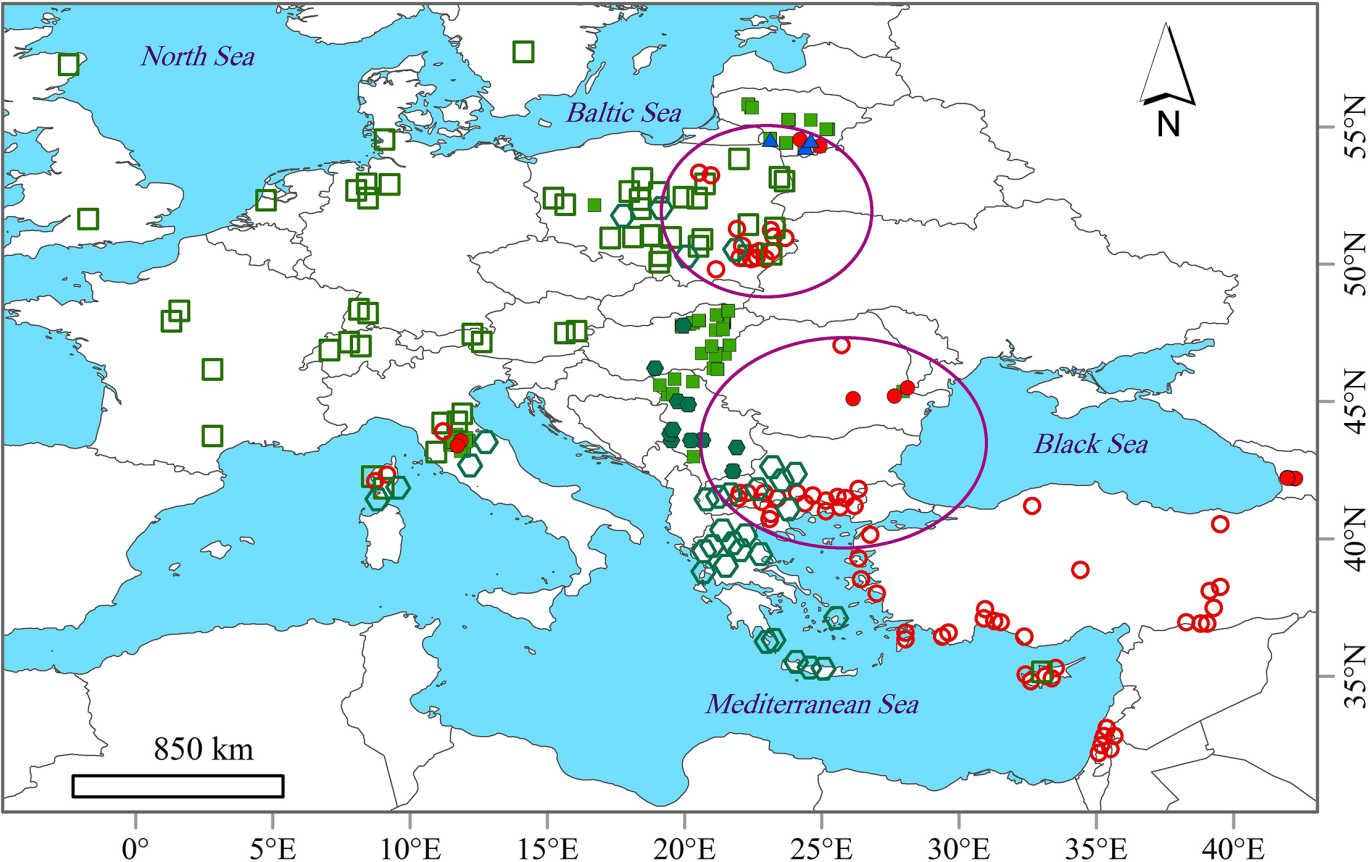
Spatial distribution of the European hares’ maternal lineages, based on the 358-bp mtDNA control region, resulting when combining sequence data from GenBank (S1 Table) and the present study. Squares and polygons indicate the Central European and South-East European subclades, respectively, in the European lineage. Circles and triangles indicate the Anatolian/Middle Eastern lineage and Mountain hare (*L*. *timidus)*, respectively. Ellipses depict the two discovered contact zone areas between brown hare lineages in South-Eastern and North-Eastern Europe. Filled geometric shapes indicate the geographic location of the sampling sites in this study. Colours of the geometric shapes are in accord with clades/lineages; light green: Central European, dark green: South-East European, red: Anatolian/Middle Eastern, blue: Mountain hare.

A total of 137 legally hunted, unprotected adult brown hares were sampled in seven countries (Hungary, Poland, Serbia, Lithuania, Romania, Georgia, Italy; Fig 1, and see S1 Table) between 2010 and 2015. Also, three mountain hares have been accidentally collected along with our samples. No animals were killed for the purposes of this research.

All tissue samples were stored in 96% ethanol at -4°C. Hair follicles samples were kept in individually registered nylon or paper bags and stored at -4°C until the laboratory analysis. Total DNA was extracted using the E.Z.N.A. Tissue DNA Kit (Omega Bio-Tek, USA), the High Pure PCR Template Preparation Kit (Roche, USA) and standard FAO protocol. DNA concentrations were evaluated spectrophotometrically and visually by standard agarose gel electrophoresis.

Different regions of the mitochondrial DNA were amplified. PCR protocols and primers (Le.H-Dloop_F, Le.L-Dloop_R [15] for the control region (CR) and LepCyb2L_F, LepD2H_R [4] for cytochrome *b* (cyt *b*) + tRNA-Thr + tRNA-Pro + control region) were used to the amplification. PCRs were carried out in a total volume of 25 μl, using the following sequence of steps: denaturation at 94°C for 5 minutes, followed by 35 cycles of amplification 94°C for 1 minute, 60°C for 1 minute and 72°C for 1 minute, and a final step at 72°C for 5 minutes. The forward sequencing reaction was performed by Macrogen Europe (The Netherlands).

In addition, previously published sequences of the species were downloaded from the GenBank (S1 and S2 Tables).

### Ethics statement

Animals were not shot for the purpose of this study. The study did not involve the collection of samples from live animals. An ethics statement was not required. Samples from the different countries were obtained from licensed collaborators and licensed hunters who took samples following their regulations in brown hare management.

### Sequence analysis

Two datasets were created from the sequences. The first dataset comprised 137 CR mtDNA sequences with a total length of 358 bp. The second dataset comprised 105 concatenated sequences cyt *b* + tRNA-Thr + tRNA-Pro + CR, with a total length of 916 bp after alignment. Alignment was performed using Seqscape 2.6 (Applied Biosystems) and ClustalW in MEGA 6 [27], respectively. The aligned sequences were deposited in GenBank with the Accession numbers: MG865671-MG865724 for CR and MG841060- MG841112 for the cyt *b* + tRNA-Thr + tRNA-Pro + CR region (S1 and S2 Tables). The European Rabbit (*Oryctolagus cuniculus*) (GenBank: AJ001588) [28] was used as an outgroup for the phylogenetic analyses. DAMBE 6 [29] was used to analyze substitution saturation.

The number of polymorphic sites, haplotype diversity, nucleotide diversity, average number of nucleotide differences for each location and number of haplotypes were estimated with DnaSP 5.10 [30]. The best-fitting partitioning scheme and nucleotide substitution model were selected using the Bayesian information criterion (BIC) and the corrected Akaike Information Criterion (AICc) implemented in PartitionFinder 2.1.1 [31].

Bayesian inference (BI) was performed using BEAST v2.3 [32] with 40,000,000 generations of Monte Carlo Markov chains (MCMC), sampling every 100 generations. Maximum likelihood (ML) analyses were implemented in IQ-TREE 1.6 [33] with 10,000 bootstrap steps. Additionally, MEGA 6 [27] was used to construct a neighbour-joining (NJ) phylogenetic tree, applying the pairwise distance data and p-distance model with 10,000 bootstrap replications. Furthermore, median-joining networks [34] among haplotypes were inferred using PopART 1.7 [35].

Fu’s *FS* [36] and Tajima’s *D* [37], performed in Arlequin 3.5 [38], were employed to assess the demographic history and to examine hypotheses of selective neutrality [39]. The significance of these tests was calculated using 10,000 permutations. The hierarchical analysis of molecular variance (AMOVA) and fixation index were implemented with 10,000 iterations using Arlequin 3.5 [38] to evaluate levels of population structure. The aim of the AMOVA analysis was to examine whether genetic variation was significantly structured among different haplogroups. *Φ*_*ST*_ can provide an estimate of the genetic differentiation among populations in order to make inferences of past demographic changes.

To estimate the presence of genetic clusters (populations) within the sequences of *L*. *europaeus* and *L*. *timidus* (or introgressed individuals), we used Bayesian Analysis of Population Structure (BAPS) v6 [40–41] implementing the method of “clustering for linked loci” with two independent runs and K = 10 repetitions. To assess introgression occurring within the populations of these two species, we performed the method of “admixture based on mixture clustering” implemented in BAPS. To provide a correct assessment of population genetic structure, it is recommended to use the admixture models, because these models are robust to an absence of admixture in the sample; reciprocally, models without admixture are not robust to the inclusion of admixed individuals in the sample [42].

## Results

### MtDNA control region sequences (358 bp)

The substitution saturation test based on both datasets (916 bp and 358 bp sequences) revealed that the base substitutions did not reach saturation, and these datasets were suitable for phylogenetic analyses.

For the 358 bp control region, 137 samples were sequenced from Central-Eastern Europe (S1 Table). Additional sequences from Europe and the Middle East published in GenBank were included in the analyses, yielding a dataset comprising a total of 447 sequences and 259 haplotypes (S1 Table). A total of 52 haplotypes were identified among the 137 new sequences, including 40 novel haplotypes and 12 previously reported haplotypes.

**Fig 2:**
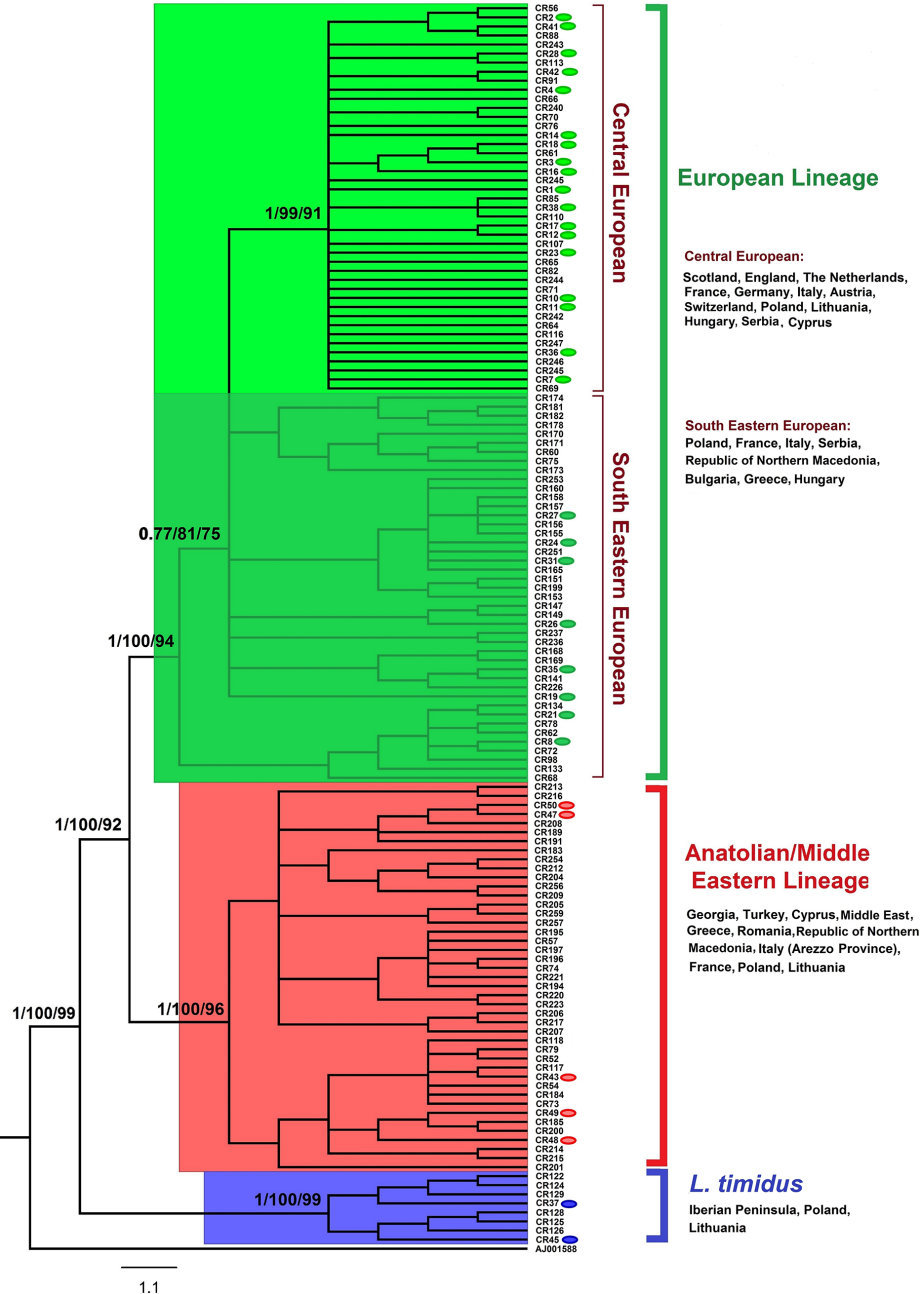
Phylogenetic relationships of brown hare from Central-Eastern Europe with other brown hares, based on the 358-bp mtDNA control region sequences and rooted with *Oryctolagus cuniculus* (AJ001588). The numbers on the branches are posterior probabilities in the Bayesian inference and bootstrap support in maximum likelihood and neighbour-joining. Colored ovals represent haplotypes identified in the current study. The branches within blue rectangular include mountain hare sequences or introgressed haplotypes of this species in other hare species. For detailed information on haplotypes see S1 Table.

The phylogenetic analyses (BI, ML, and NJ trees) yielded relatively identical topologies, indicating that among 137 selected haplotypes from the dataset (447 individuals) two lineages were identified (Fig 2).

**Fig 3:**
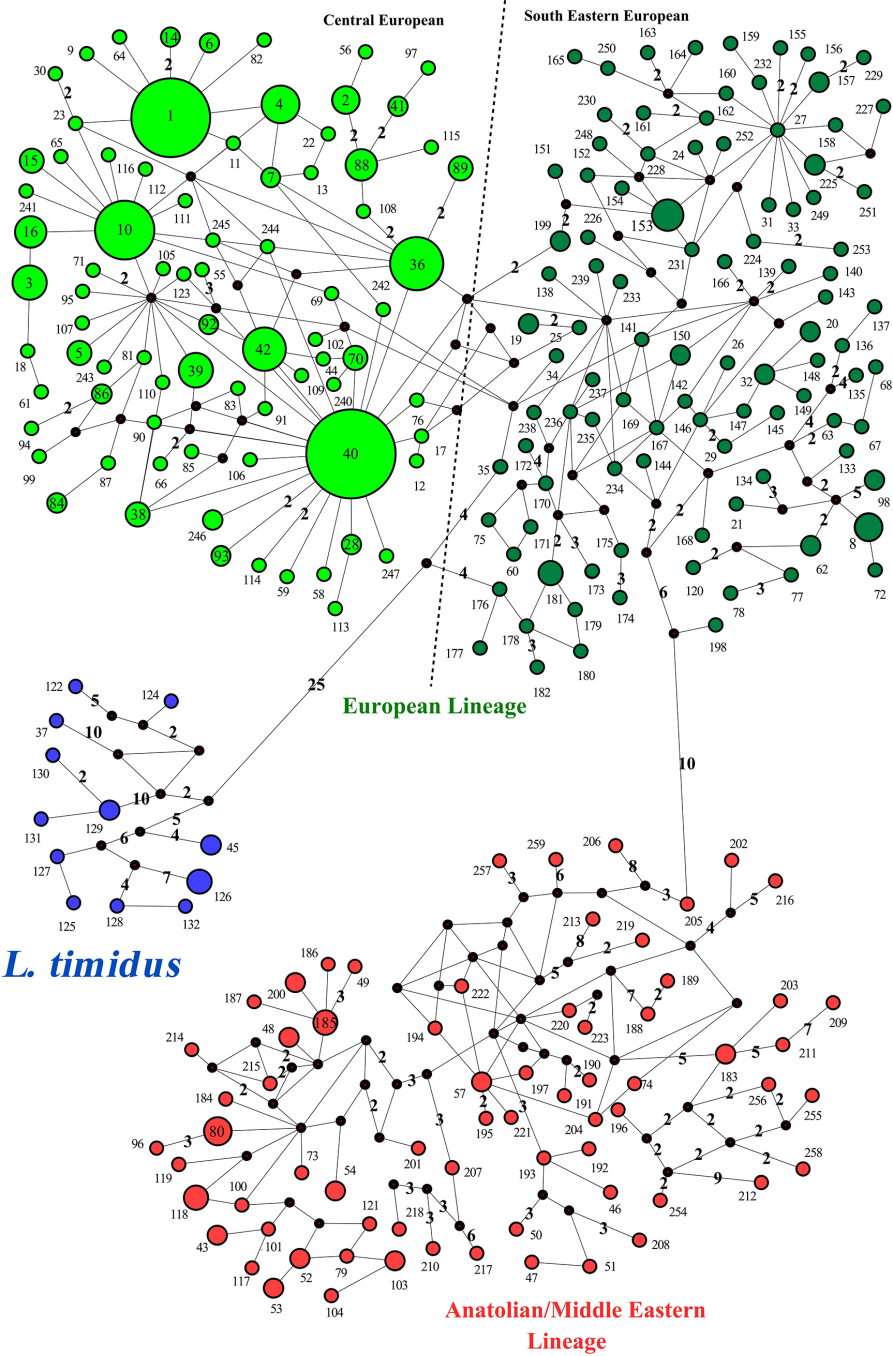
Median joining network of brown hare from Central-Eastern Europe and other brown hares, based on the 358-bp mtDNA control region. The numbers on the haplotypes (1–259) are the same haplotype codes (CR1-CR259) as in Fig 2 and S1 Table. Dark circles are connecting nodes (i.e. putative undetected haplotypes). Blue circles include mountain hare sequences or introgressed haplotypes of this species in other hare species.

The MJ network analysis (Fig 3) also supported the clusters distinguished in the phylogenetic trees. The first lineage, European (EUR), was divided into two phylogeographically distinct subclades: Central European (CE) and South-East European (SEE).

The subclade CE was mostly distributed across various regions of Central Europe, Scotland, England, the Netherlands, France, Germany, Italy, Austria, Switzerland, Poland, Lithuania, Hungary and Northern Serbia (Fig 1). However, two individuals belonging to the subclade were found in Eastern Romania and Southern Serbia. Also, one brown hare from Cyprus (Cyprus 4 in S1 Table) clustered within CE (falling into haplotype CR40, S1 Table). Haplotype CR40 along with haplotypes CR1 and CR10 was the most common haplotype in the subclade CE and was shown to inhabit more than one region in Europe (Fig 3). Haplotype CR40 was identified as the most abundant (38 individuals) and central haplotype in the subclade, and was observed across Northern Europe, from Lithuania to Poland, Germany, France, England, and Scotland. Haplotype CR1 was observed in Poland, Hungary, Romania, Serbia, and Italy, whereas haplotype CR10 was observed in Lithuania, Poland, Hungary, Serbia, Austria, Italy and France. The subclade SEE predominantly occurred in South-Eastern Europe including Bulgaria, Greece, Republic of Northern Macedonia and Serbia (Fig 1). However, individuals belonging to this subclade were also present in Hungary, Poland, Central Italy and France (Corsica Island) (Figs 1 and 2, S1 Table). Haplotypes in SEE were mostly specific to relatively limited spatial distributions (Fig 3). However, three haplotypes belonging to this subclade were recorded over a larger geographical range: CR8 (Hungary and Italy), CR32 (Serbia and Italy) and CR62 (Italy and Poland). Phylogenetic analyses revealed no shared haplotype between the subclades in this lineage.

The second cluster, the Anatolian/Middle Eastern lineage (AME) was predominantly present in Georgia, Turkey and the Middle East, and was also found in Lithuania, Poland, Romania, North-Eastern Greece, Republic of Northern Macedonia, Italy and France (Corsica Island) (Fig 1). Haplotypes in this lineage were mostly restricted to small geographic ranges. However, within AME, haplotypes CR52, CR53, and CR54 were recorded both in Romania and Italy, but haplotypes CR57 (Italy and Republic of Northern Macedonia) and CR200 (Turkey and Cyprus) were also found in distant localities (Figs 1, 2 and 3).

### MtDNA cytochrome b, tRNA-Thr, tRNA-Pro and control region (916 bp)

**Fig 4:**
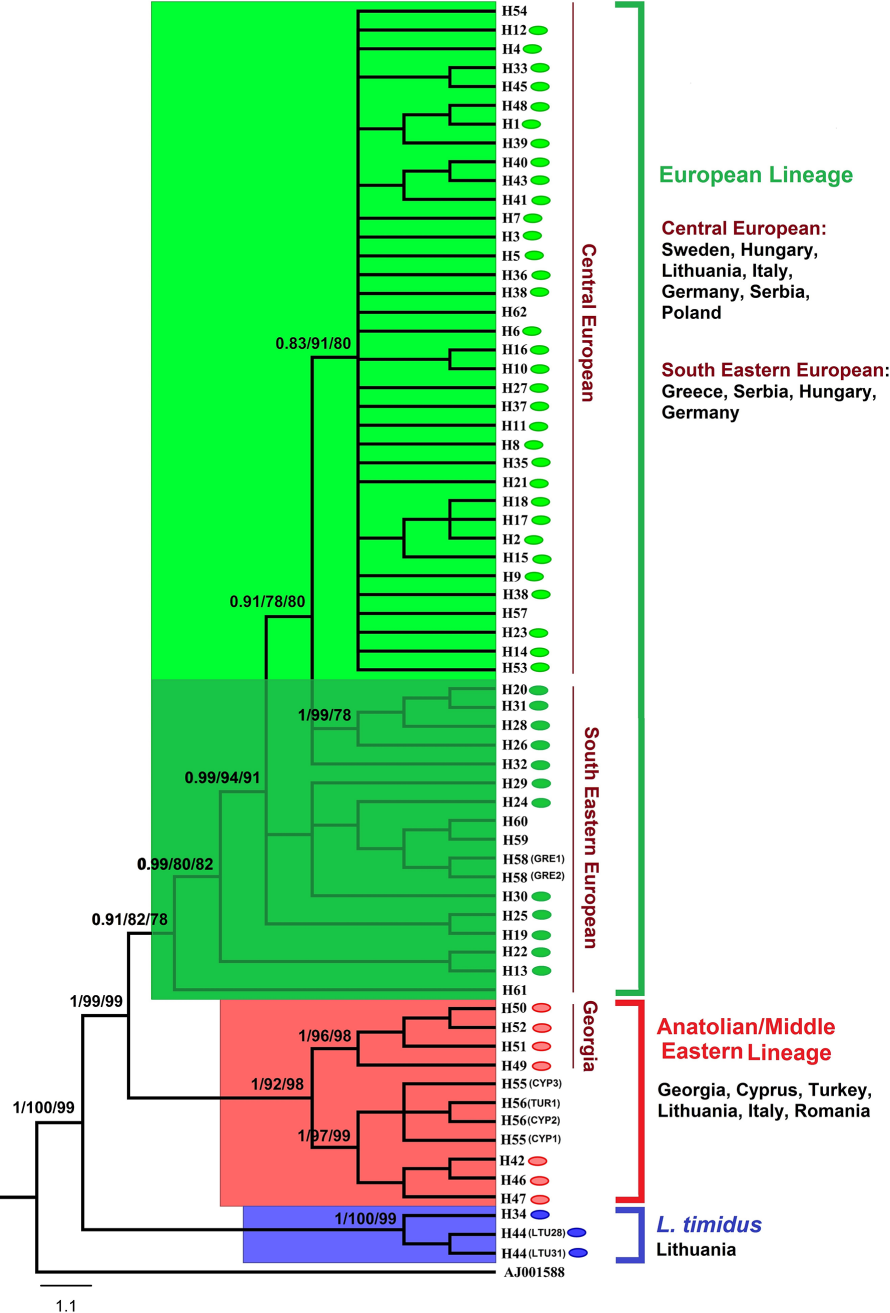
Phylogenetic relationships of brown hare from Central-Eastern Europe with other brown hares, based on the 916-bp mtDNA sequences (cyt *b* + tRNA-Thr + tRNA-Pro + control region) and rooted with *Oryctolagus cuniculus* (AJ001588). The numbers on the branches are posterior probabilities in the Bayesian inference and bootstrap support in maximum likelihood and neighbour-joining. Colored ovals represent haplotypes identified in the current study. For detailed information on haplotypes see S2 Table.

**Fig 5:**
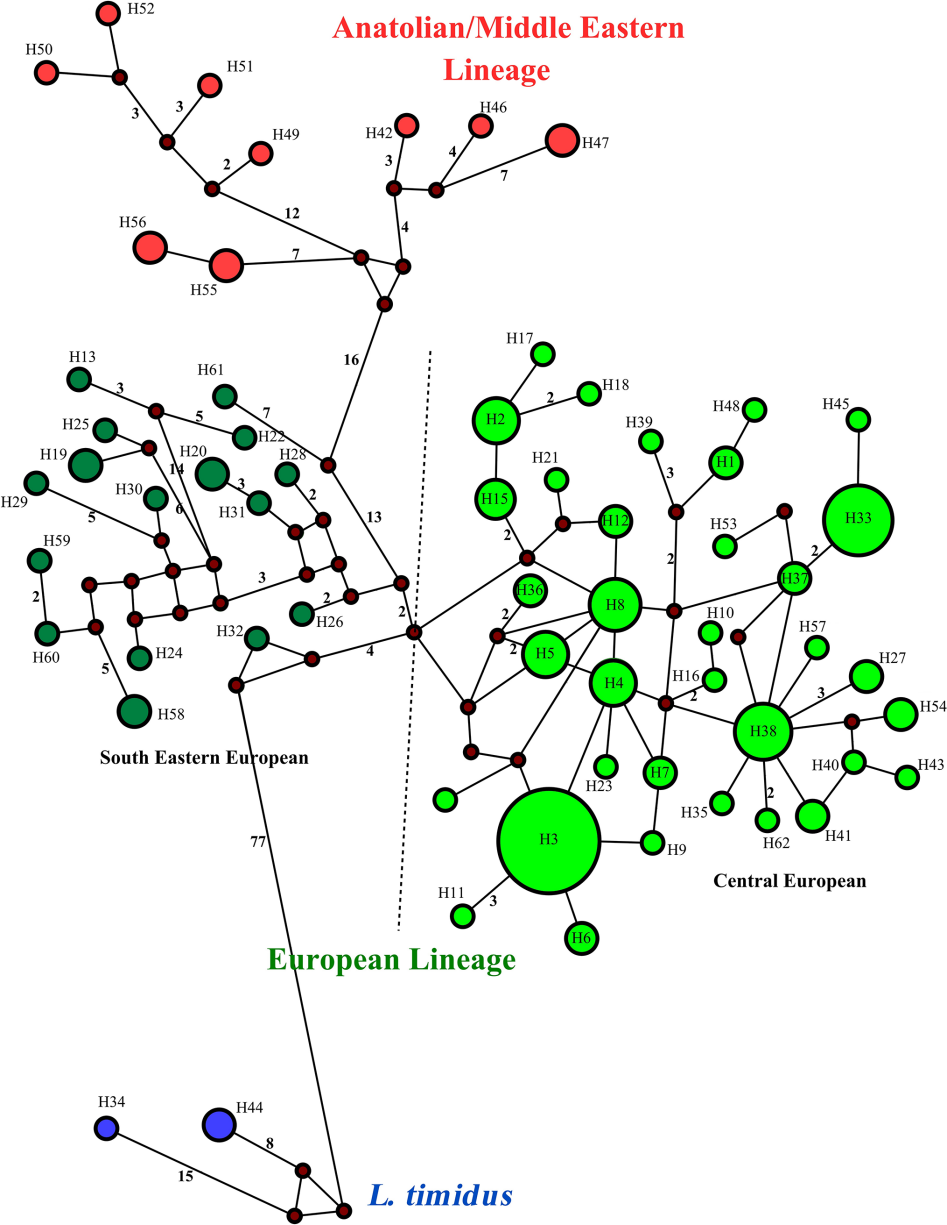
Median joining network of brown hare from Central-Eastern Europe and other brown hares, based on the 916-bp mtDNA sequences (cyt *b* + tRNA-Thr + tRNA-Pro + control region). For detailed information on haplotypes see Fig 4 and S2 Table. Dark circles are connecting nodes (i.e. putative undetected haplotypes).

Phylogenetic analyses of the control region revealed two major lineages in Central-Eastern Europe, with contact zones discovered in the geographic range (Fig 1). To obtain a better assessment of phylogeographic structure, we sequenced the additional fragments cyt *b* (426 bp), tRNA-Thr (66 bp) and tRNA-Pro (66 bp) of 105 brown hares from Italy, Hungary, Serbia, Georgia, Romania, Poland and Lithuania (S2 Table). Sixteen additional sequences belonging to brown hares from Germany, Sweden, Poland, Greece, Turkey and Cyprus available in GenBank were also added to the alignment (S2 Table). Finally, a total dataset comprising 124 sequences (916 bp fragment of mtDNA), corresponding to a total of 62 haplotypes was used for phylogenetic analysis. According to this longer fragment, and in accordance with control region sequences, the brown hare population in Central-Eastern Europe is divided into the same two distinct phylogeographic lineages (EUR and AME) (Figs 4 and 5).

Furthermore, brown hares belonging to the lineage EUR fall into two subclades, the same CE and SEE as in the first dataset. The contact zones among all lineages and subclades were identified in the same geographic ranges as in Fig 1.

A total of 51 haplotypes was found throughout Central-Eastern Europe. Moreover, 50 novel haplotypes and only one previously reported haplotype were detected among them. The genetic statistics for the sequenced brown hares in this study are displayed in Table 1.

**Table 1:** Comparison of genetic statistics for the brown hares sequenced in this study, originating from Central-Eastern Europe, based on the 916-bp mtDNA sequences (cyt *b* + tRNA-Thr + tRNA-Pro + control region).

Group	*n*	*h*	*Hd* (SD)	*Pi* (SD)	*K*	*P*	Fu’s *FS*	Tajima's *D*
Central European	83	32	0.927(0.019)	0.0051(0.0003)	4.71	41	-15.340**	-1.455*
South-East European	14	12	0.978(0.035)	0.0153(0.0021)	14.14	52	-1.567	-0.593
Anatolian/Middle Eastern	8	7	0.964(0.077)	0.0198(0.0029)	18.32	40	-0.607	0.623

*n*, number of individuals; *h*, number of haplotypes; *Hd*, haplotype diversity; SD, Standard Deviation; *Pi*, nucleotide diversity (per site); *K*, average number of nucleotide differences; *P*, variable (polymorphic) sites. Statistical significance

*p<0.05, Statistical high significance

**p<0.01.

High haplotype diversity values and relatively low-moderate nucleotide diversity were obtained for brown hares of the study populations. The lineage AME (only for Fu’s *FS*) and both the subclades of lineage EUR presented negative values for Tajima’s and Fu’s neutrality tests, but only the outcome for the Central European subclade was found significant (*D* = -1.455, *P* = 0.045; *FS* = -15.34, *P* = 0.00) (Table 1). Thus, this subclade is likely to have undergone a recent population expansion. Results of the AMOVA revealed that the among-haplogroups component of variance (67.59%) was higher than the variation within haplogroups (32.41%) (Table 2). According to the fixation index a significant genetic structure among all haplogroups was also observed (*Φ*_*ST*_ = 0.676, *P* = 0.00) (Table 2).

**Table 2:** AMOVA results for three major haplogroups (AME, SEE and CE) of brown hare originating from Central-Eastern Europe, based on the 916-bp mtDNA sequences (cyt *b* + tRNA-Thr + tRNA-Pro + control region).

Source of variation	*d*.*f*.	Percentage of variation	Fixation index (*Φ*_*ST*_)	*p-value*
Among haplogroups	2	67.59	0.676	*p*<0.000
Within haplogroups	101	32.41		
Total	103			

**Fig 6:**
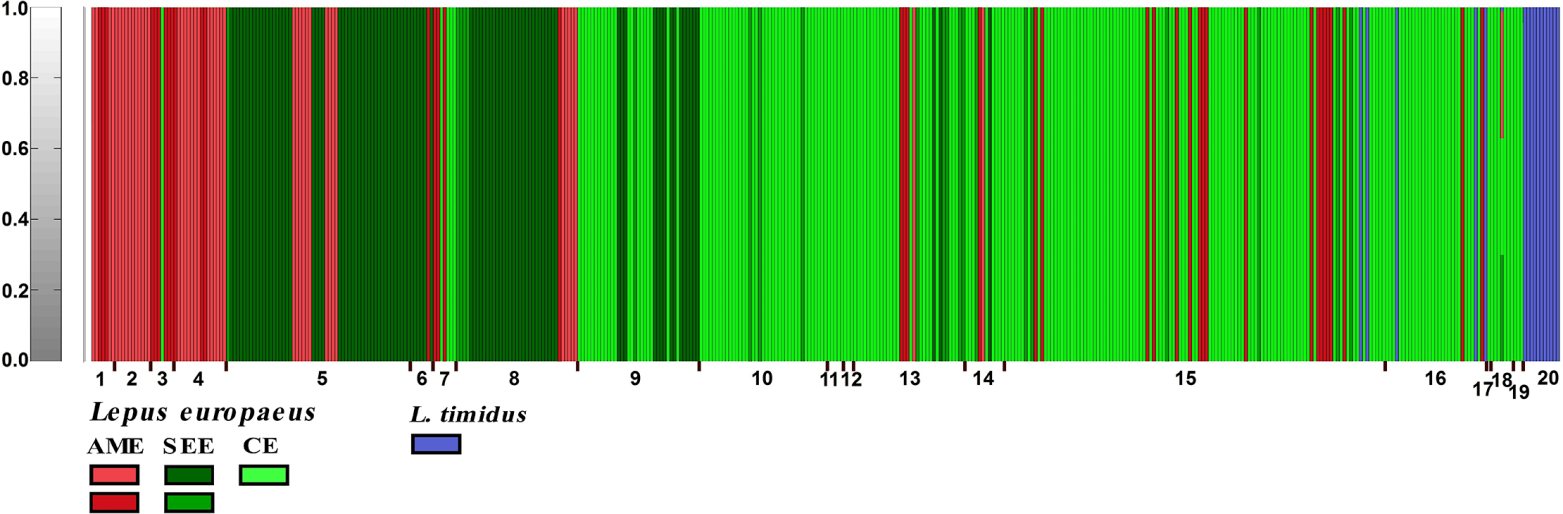
Bayesian clustering analysis of 358-bp mtDNA control region sequences from brown hares (*L*. *europaeus*) and mountain hares (*L*. *timidus* and introgressed haplotypes of this species in other hares) as implemented in BAPS v6. resulting in K = 6. We detected 5 clusters within major lineages of *L*. *europaeus*; 2 and 3 clusters within lineages AME and EUR (SEE = 2 clusters; CE = 1 cluster), respectively. Also, *L*. *timidus* and introgressed individuals were assigned to one cluster. Numbers 1 to 20 are the localities of sequence data from our study and others (see S1 Table): 1. Georgia; 2. Middle East; 3. Cyprus; 4. Turkey; 5. Greece; 6. Bulgaria; 7. Romania; 8. Republic of Northern Macedonia; 9. Serbia; 10. Hungary; 11. Austria; 12. Switzerland; 13. Italy; 14. France; 15. Poland; 16. Lithuania; 17. Sweden; 18. Germany; 19. The Netherlands, England and Scotland; 20. Iberian Peninsula.

The analysis performed with BAPS v6 separated *L*. *europaeus* and *L*. *timidus* (and introgressed mountain hare in other hare species) with K = 6 (ln(P) = −8954.5009). This analysis assigned sequences from *L*. *europaeus* to five genetic clusters, and *L*. *timidus* to only one cluster (Fig 6). Within *L*. *europaeus*, sequences belonging to lineage AME and subclade SEE (lineage EUR) were each assigned to two clusters, whereas individuals belonging to subclade CE (lineage EUR) fell into one cluster.

## Discussion

Previous studies estimated phylogenetic relationships among brown hare populations in Europe and the Middle East, where insufficient sampling left a relatively large gap in several geographic ranges, especially in Central-Eastern Europe. This information gap has prevented the delineation of a comprehensive picture of genetic diversity and phylogeographic structure of the species. European brown hares have been classified to two major lineages, European (EUR) and Anatolian/Middle Eastern (AME) [6, 15, 17–18] that co-exist in Republic of Northern Macedonia, North-Eastern Greece and Bulgaria [6, 10, 15]. In this study, we presented a relatively comprehensive dataset on mtDNA cytochrome *b*, tRNA-Thr, tRNA-Pro and control region fragments (a total of 916 bp) of brown hares in Central-Eastern Europe, where two datasets were used in the genetic analyses; the first dataset included a 358-bp control region sequence, whereas the second dataset covered a concatenated sequence of mtDNA fragments (the 916-bp sequence).

Our findings revealed a high genetic diversity within the 916-bp mtDNA sequence (105 new sequences, 51 haplotypes) of brown hares from Central-Eastern Europe, where 50 haplotypes were reported for the first time (Table 1). Phylogenetic analyses revealed two major lineages of brown hare in the study area, based on a combination of our sequences and previously published sequences (S1 and S2 Tables) for both datasets: (i) AME, which comprises individuals from Georgia, Anatolia, the Middle East and also includes some hares living in South-Eastern, North-Eastern and Central Europe, and (ii) EUR, which includes hares from Central, South-Eastern, Eastern and Northern Europe. In accordance with others [6, 15], the EUR lineage is subdivided into two well-supported subclades, Central European (CE) and South-East European (SEE).

The significant genetic structure among brown hare haplogroups from Central-Eastern Europe was well supported by *Φ*_*ST*_ and AMOVA (Table 2). The fixation index is a standard measure, which gives an estimate of the degree of genetic differentiation among and within populations/haplogroups [43]. In fact, the analyses demonstrated that partitioning into the major haplogroups explains 67.59% of the overall mtDNA variability and corresponds to a highly significant fixation index (*p*<0.000). The female philopatry of brown hares [16, 44] could have resulted in the formation of multigenerational matrilineal assemblages that are geographically structured [45].

The population structure determined by BAPS v6 partially described diversity allocation between clusters based on the control region mtDNA sequences. BAPS is known to be relatively highly efficient in identifying hidden population structures [46]. The analysis revealed five genetic clusters within the populations of *L*. *europaeus* and only one cluster within *L*. *timidus* (and introgressed) sequences. Within *L*. *europaeus*, individuals belonging to the major lineage AME were assigned to two clusters: (i) cluster 1, which includes brown hares from Georgia, Turkey, Cyprus, Bulgaria, Romania, Republic of Northern Macedonia, Central Italy, France (Corsica Island), Poland and Lithuania; (ii) cluster 2, which comprises brown hares living in the Middle East, Georgia, Turkey, Greece, Republic of Northern Macedonia, Central Italy and France (Corsica Island). Sequences belonging to subclade SEE (lineage EUR), within *L*. *europaeus*, were divided to two clusters: (i) cluster 1, including the sequences from Greece, Republic of Northern Macedonia, Serbia, Hungary, Central Italy, France (Corsica Island), Germany and Poland; (ii) cluster 2, which includes individuals from Greece, Bulgaria, Republic of Northern Macedonia, Serbia, Central Italy and France (Corsica Island). It is interesting that all genetic clusters of brown hare are present in Central Italy and France (Corsica Island) (Fig 6).

Our findings revealed some slight introgression of individual haplotypes from *L*. *timidus* into *L*. *europaeus* only in one sample (GER4 in S1 Table) from Germany (Fig 6). Extensive introgression mtDNA and nuclear genes of mountain hare into other hares has been reported in previous studies (e.g., [47–48]). The introgression of individual genotypes among populations potentially could have resulted from recent genetic hybridization or incomplete lineage sorting of ancestral variation.

The contact zones among the two major lineages (and two subclades belonging to lineage EUR), interestingly, were discovered in two large areas in Central-Eastern Europe, encompassing South-Eastern (Republic of Northern Macedonia, North-Eastern Greece, Bulgaria and Romania) and North-Eastern (Lithuania and North-Eastern Poland) Europe (Fig 1). While the sympatric distribution of haplotypes of lineages EUR and AME in Republic of Northern Macedonia, North-Eastern Greece and Bulgaria had already been shown by others [6, 10, 15], other overlapping distributions are characterized here for the first time. However, the region comprising Thrace and Bulgaria, which probably extends into Turkish Thrace and maybe into Anatolia is a well-known hybrid zone of Europe [5] for species that were restricted to refuge areas in the Southern Balkans and Anatolia during the Pleistocene cold stages [15].

Based on the combined analyses of our sequences and those of others [15; Strzala et al. unpublished, direct submission to GenBank), Polish brown hares harbour haplotypes of both lineages (and the two EUR subclades). Whereas lineage EUR (mostly the subclade CE) is widespread and predominant in Poland, another lineage is only found in the eastern part of the country. Brown hares living in Western Romania fall into the European lineage (subclade CE), whereas individuals from Eastern Romania also show haplotypes of lineage AME. Overall, our data reveal overlapping EUR and AME haplotypes both in Romania and Lithuania.

Brown hares inhabiting Georgia exhibited high genetic diversity (dataset 1: 7 individuals, 6 novel haplotypes; and dataset 2: 4 individuals, 3 new haplotypes), but only within the lineage AME. Thus, based on our data, extending the contact zone to Georgia and the Middle East, as speculated by others [6, 15] is not justified. It is interesting that among the sequences previously reported from Cyprus [15, 17], one brown hare (CYP4, listed in S1 and S2 Tables; published by [17]) shared a common haplotype (CR40 that distributes across Northern Europe; see Fig 3 and S1 Table for detailed information) of European lineage origin (subclade CE). However, the haplotype was found outside the range of Northern Europe only in Cyprus.

We consider human-mediated translocations for these introgressions, as has been widely confirmed for both recent and historic times [15, 49–50]. However, more extensive samplings, especially in Eastern Europe, Balkans, north of the Black Sea and Anatolia, may reveal important phylogeographic signatures.

Our data confirm the presence of both subclades (CE and SEE) belonging to the lineage EUR in Hungary and Serbia. Whereas haplotypes belonging to SEE are predominant in Southern and Central Serbia, the unique sequences of CE are predominantly found in Hungary and Northern Serbia. Moreover, a recent study reported one haplotype belonging to AME among brown hares from Northern Serbia as a possible consequence of human-mediated translocations [18].

According to the combined analysis of our sequences and those of others [51], haplotypes belonging to lineages EUR (both subclades CE and SEE) and AME are present in Italy. Nevertheless, haplotypes belonging to CE are predominant in this country. The European brown hare is a major game species in Europe [52], and different populations of the species have been introduced in different areas, mostly for hunting. Thus, this presence of AME is also probably due to human-mediated translocations, as reported in other studies (e.g., [51]. Furthermore, the occurrence of *L*. *europaeus* in Corsica is recent and artificial, as it is known that different species of hares have been introduced in the region up to this day [53]. Overall, the presence of both major lineages (and the European subclades) of brown hare in Corsica could be the result of several human-mediated introduction events from different origins [54]. Likewise, a contact zone between mountain hares (*L*. *timidus*) and brown hares can be observed in Lithuania, as recorded in different populations of brown hares [9, 48,55–57].

The network result, in accordance with Stamatis et al. [6], showed that there are relatively close relationships between some haplotypes belonging to CE and several haplotypes from SEE (Fig 3). This finding indicates that one haplotype of the first subclade is only connected by one, so far undetected, haplotype, to another haplotype from the second subclade. However, the network analysis based on the longer sequence (916 bp) (Fig 5) does not provide strong support for this hypothesis. Overall, the close phylogenetic relationships between the two subclades SEE and CE in large geographic ranges of Europe support the idea that the brown hare colonized the current spatial ranges, when ecological conditions in these areas became suitable for the species after the Last Glacial Maximum [6, 58]. Also, the presence of a large number of unique haplotypes in South-Eastern Europe (the Balkans) and Anatolia is evidence for maintenance of a high proportion of the pre-glacial brown hare diversity in these areas during at least the last glacial period. Other studies have demonstrated the high intraspecies diversity of brown hare in these areas [6, 15, 18].

We discovered large contact zones for brown hares in several countries of Central-Eastern Europe. These findings support the existence of probable glacial refugia during the LGM in some of these areas (especially in Southern Europe), where the refugial populations probably underwent genetic differentiation [8], resulting in a number of genetic clusters. Following the retreat of the glaciers, the genetically isolated populations colonized Europe and formed secondary contact zones [59]. Our findings are in accordance with others [6–8, 15] who suggest the post-glacial population expansion scenario from southern refugia (such as Iberia, Italy and the Balkans, as well as Asia Minor and the Caspian/Caucasus region). Other studies [18] provide evidence for the hypothesis of an Anatolian population range expansion of the brown hare into south-eastern and south-central areas of the Balkans, which has likely acted as a potentially important source in the pattern of gene flow to southern, central and northern areas of the Balkan Peninsula. Furthermore, it is suggested that colonization of the central and western parts of Europe by brown hares started from the Northern Balkans in a postglacial expansion. However, the Balkans were the most important source of European populations, due to the lack of major geographical barriers limiting a northward expansion, compared to the Alps and the Pyrenees for the Italian and the Iberian refugia, respectively [7]. Several authors described the existence of introgression of Anatolian mtDNA in Bulgarian brown hares which most likely result of hunting management practices in recent time [6, 15, 18, 49]. The colonization pattern of Central and Northern Europe from the Balkan Peninsula has also been proposed for other species such as the marbled white butterfly (*Melanargia galathea*) [60] and the wild boar (*Sus scrofa*) [61].

Our data, in combination with additional ones [6, 17, 48], indicate phylogenetically close relationships among brown hares throughout Central and Northern Europe, where a common haplotype (CR40 in Fig 3 and S1 Table) was identified. Furthermore, other shared haplotypes (e.g., CR1 and CR10) were found from the east (Lithuania, Romania, Serbia) to central (Poland, Hungary, Austria) and west (Italy and France) across Europe. The findings suggest that source regions for the origin of northern, western, and central populations of brown hare are probably situated in Eastern or Southern Europe. High variability of mtDNA in these probable sources support the hypothesis of gene flow in a northward and westward expansion of the identified contact zones, as Stamatis et al. [6] proposed the gene flow from north-western populations of Greece into Central Italy via a land bridge between the Balkans and the Italian peninsula at the end of the Pleistocene and the Holocene. Also, Stamatis et al. [6] suggested the probable pattern of gene flow northward from Italy to Switzerland and Austria, after the retreat of the southern alpine glaciers. Several studies suggested the postglacial colonization of Central and North-Western continental Europe by the brown hare of the Balkans [6, 15, 18]. Others [62] supported the postglacial recolonization of Central Europe by the Italian populations.

The existence of AME haplotypes in South-Eastern Europe support a sudden expansion of this lineage to Europe during the late Pleistocene via the Bosphorus land bridge that disappeared only ca. 8000 years ago with the rise of the sea level [18, 63] or some Greek islands when they were still connected to Anatolia in the late Pleistocene [15]. On the other hand, the presence of a genetic break at the border between Anatolia and the surrounding regions has been reported in different species [64].

Also, our data confirm the presence of AME haplotypes in North-Eastern Europe, indicating the gene flow from Anatolian/Middle Eastern brown hares into Eastern and North-Eastern Europe via west of the Black Sea or other post-glacial colonization routes, especially east of the Black Sea. Alternatively, the existence of some haplotypes out of their lineage's original homeland may be due to recent translocations. Indeed, Kasapidis et al. [15] described that the brown hares living in some Eastern Mediterranean islands (such as Crete and Cyprus) have probably been introduced by humans because these islands were cut off from the mainland more than 2.5 million years ago.

Neutrality tests were negative for the lineages and subclades (except in AME for the value of Tajima's *D*), but only the subclade CE showed a significant negative value, indicating a significant excess of rare haplotypes suggesting that the population is not under mutation-drift equilibrium due to sudden expansion [45, 65]. Also, the star-like connection pattern of haplotypes (CR1, 10, 27, 36, 40, 57, and 167 in Fig 3; and H3, 8 and 38 in Fig 5) gives support to the hypothesis of population expansion [66]. Some of these haplotypes are the central and most abundant ones and are widely distributed in the study area. Thus, it is highly likely that the common and central haplotypes are ancestral haplotypes. Moreover, the patterns of high haplotype diversity along with relatively low nucleotide diversity (as found in this study) indicate sudden demographic expansion from a restricted area or a small effective population size in the recent past [65, 67]. In other words, this pattern suggests that the populations originate from closely related haplotypes.

## Supporting information

S1 Table Details of sequences used in the phylogenetic analyses of brown hares based on the 358 bp mtDNA control region. (XLSX)

S2 Table Details of sequences used in the phylogenetic analyses of brown hares based on the 916 bp mtDNA sequences (cytochrome *b*, tRNA-Thr, tRNA-Pro and control region). (XLSX)

## References

[1] Flux JEC, Angermann R. 1990 The hares and jackrabbits. Rabbits, hares and pikas. Status survey and conservation action plan In: Chapman J. A. & Flux J. E. C. (Eds.), *IUCN/SSC Lagomorph Specialist Group* (Vol. 1, pp. 61–94) Gland, CH & Cambridge, UK: IUCN.

[2] Ben Slimen H, Suchentrunk F, Memmi A, Sert H, Kryger U, Alves PC, et al Evolutionary relationships among hares from North Africa (*Lepus sp.* or *Lepus spp.*), cape hares (*L. capensis*) from South Africa, and brown hares (*L. europaeus*), as inferred from mtDNA PCR‐RFLP and allozyme data. Journal of Zoological Systematics and Evolutionary Research. 2006 44(1): 88–99. 10.1111/j.1439-0469.2005.00345.x

[3] Ben Slimen H, Suchentrunk F, Stamatis C, Mamuris Z, Sert H, Alves PC, et al Population genetics of cape and brown hares (*Lepus capensis* and *L. europaeus*): A test of Petter's hypothesis of conspecificity. Biochemical Systematics and Ecology. 2008 36(1): 22–39. 10.1016/j.bse.2007.06.014

[4] Pierpaoli M, Riga F, Trocchi V, Randi E. Species distinction and evolutionary relationships of the Italian hare (*Lepus corsicanus*) as described by mitochondrial DNA sequencing. Molecular Ecology. 1999 8(11): 1805–1817. 10.1046/j.1365-294x.1999.00766.x 10620225

[5] Hewitt GM. Post-glacial re-colonization of European biota. Biological Journal of the Linnean Society. 1999 68(1–2): 87–112. 10.1111/j.1095-8312.1999.tb01160.x

[6] Stamatis C, Suchentrunk F, Moutou KA, Giacometti M, Haerer G, Djan M, et al Phylogeography of the brown hare (*Lepus europaeus*) in Europe: a legacy of southeastern Mediterranean refugia? Journal of Biogeography. 2009 36(3): 515–528. 10.1111/j.1365-2699.2008.02013.x

[7] Taberlet P, Fumagalli L, Wust-Saucy AG, Cosson JF. Comparative phylogeography and postglacial colonization routes in Europe. Molecular Ecology. 1998 7(4): 453–464. 10.1046/j.1365-294x.1998.00289.x 9628000

[8] Hewitt G. The genetic legacy of the Quaternary ice ages. Nature. 2000 405(6789): 907–913. 10.1038/35016000 10879524

[9] Alves PC, Melo-Ferreira J, Branco M, Suchentrunk F, Ferrand N, Harris DJ. Evidence for genetic similarity of two allopatric European hares (*Lepus corsicanus* and *L. castroviejoi*) inferred from nuclear DNA sequences. Molecular Phylogenetics and Evolution. 2008 46(3): 1191–1197. 10.1016/j.ympev.2007.11.010 18178109

[10] Antoniou A, Magoulas A, Platis P, Kotoulas G. Assessing the genetic landscape of a contact zone: the case of European hare in northeastern Greece. Genetica. 2013 141(1–3): 23–40. 10.1007/s10709-013-9703-z 23381134

[11] Guberti V, de Marco MA, Riga F, Cavazza A, Trocchi V, Capucci L. Virolog and species conservation: the case of EBHSV and the Italian hare (*Lepus corsicanus* De Winton, 1898). Proceedings of V Int. Congress of European Society for Veterinary Virology, Brescia. 2000 8: 27–30.

[12] Thulin CG, Isaksson M, Tegelström H. The origin of Scandinavian mountain hares. Gibier Faune Sauvage, Game Wildlife. 1997 14(3): 463–475.

[13] Melo‐Ferreira J, Boursot P, Suchentrunk F, Ferrand N, Alves PC. Invasion from the cold past: extensive introgression of mountain hare (*Lepus timidus*) mitochondrial DNA into three other hare species in northern Iberia. Molecular Ecology. 2005 14(8): 2459–2464. 10.1111/j.1365-294X.2005.02599.x 15969727

[14] Smith AT. 2008 Conservation of endangered lagomorphs In: Alves P.C., Ferrand N. & Hacklander K (Eds.), *Lagomorph biology: evolution, ecology and conservation* (pp. 297–315). Berlin, D: Springer.

[15] Kasapidis P, Suchentrunk F, Magoulas A, Kotoulas G. The shaping of mitochondrial DNA phylogeographic patterns of the brown hare (*Lepus europaeus*) under the combined influence of Late Pleistocene climatic fluctuations and anthropogenic translocations. Molecular Phylogenetics and Evolution. 2005 34(1): 55–66. 10.1016/j.ympev.2004.09.007 15579381

[16] Mamuris Z, Moutou KA, Stamatis C, Sarafidou T, Suchentrunk F. Y DNA and mitochondrial lineages in European and Asian populations of the brown hare (*Lepus europaeus*). Mammalian Biology-Zeitschrift für Säugetierkunde. 2010 75(3): 233–242. 10.1016/j.mambio.2009.01.004

[17] Giannoulis T, Stamatis C, Tsipourlianos A, Mamuris Z. Mitogenomic analysis in European brown hare (*Lepus europaeus*) proposes genetic and functional differentiation between the distinct lineages. Mitochondrial DNA. Part A, DNA mapping, sequencing, and analysis Mitochondrial DNA Part A. 2018 1–8. 10.1080/24701394.2016.1278540 28129721

[18] Djan M, Stefanović M, Veličković N, Lavadinović V, Alves PC, Suchentrunk F. Brown hares (*Lepus europaeus* Pallas, 1778) from the Balkans: a refined phylogeographic model. Hystrix, the Italian Journal of Mammalogy. 2017 28(2): 1–8. 10.4404/hystrix-28.2–12202

[19] Sert H, Ben Slimen H, Erdoğan A, Suchentrunk F. Mitochondrial HVI sequence variation in Anatolian hares (*Lepus europaeus* Pallas, 1778). Mammalian Biology-Zeitschrift für Säugetierkunde. 2009 74(4): 286–297. 10.1016/j.mambio.2008.05.008

[20] Yu L, Li YW, Ryder O, Zhang YP. Analysis of complete mitochondrial genome sequences increases phylogenetic resolution of bears (*Ursidae*), a mammalian family that experienced rapid speciation. BMC Evolutionary Biology. 2007 7(1): 198 10.1186/1471-2148-7-198 17956639 PMC2151078

[21] Zhou T, Shen X, Irwin DM, Shen Y, Zhang Y. Mitogenomic analyses propose positive selection in mitochondrial genes for high-altitude adaptation in galliform birds. Mitochondrion. 2014 18: 70–75. 10.1016/j.mito.2014.07.012 25110061

[22] Hartl GB, Suchentrunk F, Nadlinger K, Willing R. Studies on the European hare. 47. An integrative analysis of genetic differentiation in the brown hare *Lepus europaeus* based on morphology, allozymes, and mitochondrial DNA. Acta Theriologica. 1993 38(2): 33–57.

[23] Vapa L, Obreht D, Vapa M, Selmic V. Genetic variability in brown hare (*Lepus europeaus*) populations in Yugoslavia. Zeitschrift für Jagdwissenschaft. 2002 48: 261–266. 10.1007/BF02192416

[24] Vapa L, Djan M, Obreht D, Hammer S, Suchentrunk F. Allozyme variability of brown hares (*Lepus europaeus*) from the Vojvodina (Serbia), compared to central and south eastern European populations. Acta Zoologica Academiae Scientiarum Hungaricae. 2007 53(1): 75–87.

[25] Djan M, Obreht D, Vapa L. Polymorphism of mtDNA regions in brown hare (*Lepus europaeus*) populations from Vojvodina (Serbia and Montenegro). European Journal of Wildlife Research. 2006 52(4): 288–291. 10.1007/s10344-006-0050-6

[26] Djan M, Veličković N, Stefanović M, Marković V, Vidaković DO, Vapa L. Genetic variation within and among brown hare (*Lepus europaeus* Pallas, 1778) populations in Serbia as inferred from microsatellites. Balkan Journal of Wildlife Research. 2015 2(1): 18–26. doi: 10.15679/bjwr.v2i1.22

[27] Tamura K, Stecher G, Peterson D, Filipski A, Kumar S. MEGA6: molecular evolutionary genetics analysis version 6.0. Molecular Biology and Evolution. 2013 30(12): 2725–2729. 10.1093/molbev/mst197 24132122 PMC3840312

[28] Gissi C, Gullberg A, Arnason U. The complete mitochondrial DNA sequence of the rabbit, *Oryctolagus cuniculus*. Genomics. 1998 50(2): 161–169. 10.1006/geno.1998.5282 9653643

[29] Xia X. DAMBE6: New tools for microbial genomics, phylogenetics and molecular evolution. Journal of Heredity. 2017 108(4): 431–437. 10.1093/jhered/esx033 28379490 PMC5434544

[30] Librado P, Rozas J. DnaSP v5: a software for comprehensive analysis of DNA polymorphism data. Bioinformatics. 2009 25(11): 1451–1452. 10.1093/bioinformatics/btp187 19346325

[31] Lanfear R, Frandsen PB, Wright AM, Senfeld T, Calcott B. Partition Finder 2: new methods for selecting partitioned models of evolution for molecular and morphological phylogenetic analyses. Molecular Biology and Evolution. 2016 34(3): 772–773. 10.1093/molbev/msw260 28013191

[32] Bouckaert R, Heled J, Kühnert D, Vaughan T, Wu CH, Xie D, et al BEAST 2: a software platform for Bayesian evolutionary analysis. PLoS Computational Biology. 2014 10(4): e1003537 10.1371/journal.pcbi.1003537 24722319 PMC3985171

[33] Nguyen LT, Schmidt HA, von Haeseler A, Minh BQ. IQ-TREE: a fast and effective stochastic algorithm for estimating maximum-likelihood phylogenies. Molecular Biology and Evolution. 2015 32(1): 268–274. 10.1093/molbev/msu300 25371430 PMC4271533

[34] Bandelt HJ, Forster P, Röhl A. Median-joining networks for inferring intraspecific phylogenies. Molecular Biology and Evolution. 1999 16(1): 37–48. 10.1093/oxfordjournals.molbev.a026036 10331250

[35] http://popart.otago.ac.nz/downloads.shtml.

[36] Fu YX. Statistical tests of neutrality of mutations against population growth, hitchhiking andbackground selection. Genetics. 1997 147(2): 915–925. 9335623 10.1093/genetics/147.2.915 PMC1208208

[37] Tajima F. The effect of change in population size on DNA polymorphism. Genetics. 1989 123(3): 597–601. 2599369 10.1093/genetics/123.3.597 PMC1203832

[38] Excoffier L, Lischer HE. Arlequin suite ver 3.5: a new series of programs to perform populationgenetics analyses under Linux and Windows. Molecular Ecology Resources. 2010 10(3): 564–567. 10.1111/j.1755-0998.2010.02847.x 21565059

[39] Ramírez-Soriano A, Ramos-Onsins SE, Rozas J, Calafell F, Navarro A. Statistical power analysis of neutrality tests under demographic expansions, contractions and bottlenecks with recombination. Genetics. 2008 179(1): 555–567. 10.1534/genetics.107.083006 18493071 PMC2390632

[40] Corander J, Waldmann P, Marttinen P, Sillanpää MJ. BAPS 2: enhanced possibilities for the analysis of genetic population structure. Bioinformatics. 2004 20(15): 2363–2369. 10.1093/bioinformatics/bth250 15073024

[41] Corander J, Marttinen P, Sirén J, Tang J. Enhanced Bayesian modelling in BAPS software for learning genetic structures of populations. BMC bioinformatics. 2008 9(1): 539 10.1186/1471-2105-9-539 19087322 PMC2629778

[42] François O, Durand E. Spatially explicit Bayesian clustering models in population genetics. Molecular Ecology Resources. 2010 10(5): 773–784. 10.1111/j.1755-0998.2010.02868.x 21565089

[43] Neigel JE. Is FST obsolete? Conservation Genetics. 2002 3(2): 167–73.

[44] Fickel J, Schmidt A, Putze M, Spittler H, Ludwig A, Streich WJ, et al Genetic structure of populations of European brown hare: implications for management. Journal of Wildlife Management. 2005 69(2): 760–770. 10.2193/0022-541X(2005)069[0760:GSOPOE]2.0.CO;2

[45] Ashrafzadeh MR, Kaboli M, Naghavi MR. Mitochondrial DNA analysis of Iranian brown bears (*Ursus arctos*) reveals new phylogeographic lineage. Mammalian Biology-Zeitschrift für Säugetierkunde. 2016 81(1): 1–9. 10.1016/j.mambio.2015.09.001

[46] Corander J, Marttinen P. Bayesian identification of admixture events using multilocus molecular markers. Molecular Ecology. 2006 15(10): 2833–2843. 10.1111/j.1365-294X.2006.02994.x 16911204

[47] Levänen R, Thulin CG, Spong G, Pohjoismäki JL. Widespread introgression of mountain hare genes into Fennoscandian brown hare populations. PloS One. 2018 13(1): e0191790 10.1371/journal.pone.0191790 29370301 PMC5784980

[48] Melo-Ferreira J, Boursot P, Carneiro M, Esteves PJ, Farelo L, Alves PC. Recurrent introgression of mitochondrial DNA among hares (*Lepus* spp.) revealed by species-tree inference and coalescent simulations. Systematic Biology. 2012 61(3): 367–381. 10.1093/sysbio/syr114 22201159

[49] Mamuris Z, Sfougaris AI, Stamatis C. Genetic structure of Greek brown hare (*Lepus europaeus*) populations as revealed by mtDBNA RFLP-PCR analysis: implications for conserving genetic diversity. Biological Conservation. 2001 101(2): 187–196. 10.1016/S0006-3207(01)00065-9

[50] Suchentrunk F, Ben Slimen HB, Stamatis C, Sert H, Scandura M, Apollonio M, et al Molecular approaches revealing prehistoric, historic, or recent translocations and introductions of hares (Genus Lepus) by humans. Human Evolution. 2006 21(2): 151–165. 10.1007/s11598-006-9016-7

[51] Canu A, Scandura M, Luchetti S, Cossu A, Iacolina L, Bazzanti M, et al Influence of management regime and population history on genetic diversity and population structure of brown hares (*Lepus europaeus*) in an Italian province. European Journal of Wildlife Research. 2013 59(6): 783–793. 10.1007/s10344-013-0731-x

[52] Fontanesi L, Di Palma F, Flicek P, Smith AT, Thulin CG, Alves PC, et al LaGomiCs—Lagomorph Genomics Consortium: An International Collaborative Effort for Sequencing the Genomes of an Entire Mammalian Order. Journal of Heredity. 2016 107(4): 295–308. 10.1093/jhered/esw010 26921276 PMC4888434

[53] Pietri C. 2007 Caccia e protezione delle popolazioni di lepre (*Lepus sp.*) in Corsica In: De Filippo G., De Riso L., Riga F, Trocchi V, & Troisi S. R. (Eds.), *Conservazione di Lepus corsicanus De Winton, 1898 e stato delle conoscenze* (pp. 52–62). Napoli, IT: IGF Publication.

[54] Pietri C, Alves PC, Melo-Ferreira J. Hares in Corsica: high prevalence of *Lepus corsicanus* and hybridization with introduced *L*. *europaeus* and *L*. *granatensis*. European Journal of Wildlife Research. 2011 57(2): 313–321. 10.1007/s10344-010-0430-9

[55] Alves PC, Ferrand N, Suchentrunk F, Harris DJ. Ancient introgression of *Lepus timidus* mtDNA into *L*. *granatensis* and *L*. *europaeus* in the Iberian peninsula. Molecular Phylogenetics and Evolution. 2003 27(1): 70–80. 10.1016/S1055-7903(02)00417-7 12679072

[56] Melo-Ferreira J, Alves PC, Freitas H, Ferrand N, Boursot P. The genomic legacy from the extinct *Lepus timidus* to the three hare species of Iberia: contrast between mtDNA, sex chromosomes and autosomes. Molecular Ecology. 2009 18(12): 2643–2658. 10.1111/j.1365-294X.2009.04221.x 19457181

[57] Thulin CG, Fang M, Averianov AO. Introgression from *Lepus europaeus* to *L*. *timidus* in Russia revealed by mitochondrial single nucleotide polymorphisms and nuclear microsatellites. Hereditas. 2006 143: 68–76. 10.1111/j.2006.0018-0661.01952.x 17362337

[58] Corbet GB. Relationships and origins of the European lagomorphs. Mammal Review. 1986 16(3‐4): 105–110. 10.1111/j.1365-2907.1986.tb00029.x

[59] Hewitt GM. Genetic consequences of climatic oscillations in the Quaternary. Philosophical Transactions of the Royal Society of London B: Biological Sciences. 2004 359(1442): 183–195. 10.1098/rstb.2003.1388 15101575 PMC1693318

[60] Schmitt T, Habel JC, Zimmermann M, Mueller P. Genetic differentiation of the marbled white butterfly, *Melanargia galathea*, accounts for glacial distribution patterns and postglacial range expansion in southeastern Europe. Molecular Ecology. 2006 15(7): 1889–1901. 10.1111/j.1365-294X.2006.02900.x 16689905

[61] Alexandri P, Triantafyllidis A, Papakostas S, Chatzinikos E, Platis P, Papageorgiou N, et al The Balkans and the colonization of Europe: the post‐glacial range expansion of the wild boar, *Sus scrofa*. Journal of Biogeography. 2012 39(4): 713–723. 10.1111/j.1365-2699.2011.02636.x

[62] Fickel J, Hauffe HC, Pecchioli E, Soriguer R, Vapa L, Pitra C. Cladogenesis of the European brown hare (*Lepus europaeus* Pallas, 1778). European Journal of Wildlife Research. 2008 54(3): 495–510. 10.1007/s10344-008-0175-x

[63] Gökaşan E, Demirbağ E, Oktay FY, Ecevitoğlu B, Şimşek M, Yüce H. On the origin of the Bosporus. Marine Geology. 1997 140(1–2): 183–199. 10.1016/S0025-3227(97)00022-4

[64] Bilgin R. Back to the suture: the distribution of intraspecific genetic diversity in and around Anatolia. International Journal of Molecular Sciences. 2011 12(6): 4080–4103. 10.3390/ijms12064080 21747726 PMC3131610

[65] Ojeda AA. Phylogeography and genetic variation in the South American rodent Tympanoctomys barrerae (Rodentia: Octodontidae). Journal of Mammalogy. 2010 91(2): 302–313. 10.1644/09-MAMM-A-177.1

[66] Slatkin M, Hudson RR. Pairwise comparisons of mitochondrial DNA sequences in stable and exponentially growing populations. Genetics. 1991 129(2): 555–562. 1743491 10.1093/genetics/129.2.555 PMC1204643

[67] Avise JC. 2000 *Phylogeography: the history and formation of species* Harvard University Press, MA: Cambridge.

